# Balancing Polysulfide Distribution in “Anode‐Free” Lithium–Sulfide Batteries

**DOI:** 10.1002/cssc.202501104

**Published:** 2025-10-08

**Authors:** Lennart Wichmann, Aleksei Sadykov, Pascal Seete, Bärbel Tengen, Peng Yan, Tom Boenke, Isidora Cekic‐Laskovic, Sascha Nowak, Holger Althues, Stefan Kaskel, Martin Winter, Gunther Brunklaus

**Affiliations:** ^1^ Helmholtz – Institute Münster IMD‐4 Forschungszentrum Jülich GmbH Corrensstr. 46 48149 Münster Germany; ^2^ MEET Battery Research Center, Institute of Physical Chemistry University of Münster Corrensstr. 46 48149 Münster Germany; ^3^ International Graduate School for Battery Chemistry, Characterization, Analysis, Recycling and Application (BACCARA) University of Münster Corrensstr. 40 48149 Münster Germany; ^4^ Chair of Inorganic Chemistry Technical University Dresden Bergstraße 66 01069 Dresden Germany; ^5^ Department Chemical Surface and Battery Technology Fraunhofer Institute of Materials and Beam Technology (IWS) Winterbergstraße 28 01277 Dresden Germany

**Keywords:** anode‐free, functional separators, lithium–sulfur batteries, reversibility, shuttle effect

## Abstract

Lithium–sulfide positive electrodes represent a promising alternative to established transition metal‐based positive electrodes due to enhanced specific capacity and sustainability. While positive electrodes containing elemental sulfur require a lithiated negative electrode, lithium–sulfide can serve as the lithium reservoir and thus be paired with bare copper electrodes in “anode‐free” or “zero‐excess” cell concepts. This boosts energy density and avoids handling of thin lithium metal electrodes. While promising electrochemical performance of “anode‐free” lithium–sulfide batteries has already been demonstrated, many reported cell configurations rely on nickel‐ instead of copper‐based negative electrodes, undermining the enhanced sustainability bestowed by lithium–sulfide positive electrodes. Demonstrating a continuous reaction between copper electrodes and soluble polysulfide species, two approaches are evaluated that restrict the migration of polysulfide species. While both, in situ polymerization of an electrolyte additive as well as electrospinning of a polymer layer at negative electrodes, enable reversible operation of copper‐based “anode‐free” lithium–sulfide batteries, the former approach offers notably enhanced capacity retention. Counterintuitively, the quantification of polysulfide distribution throughout the individual battery components reveals less confinement within the positive electrode as beneficial for the overall reversibility. This demonstrates that a balance between positive and negative electrode reversibility is required to advance “anode‐free” lithium–sulfide batteries.

## Graphical abstract

“Anode‐free” lithium–sulfide batteries represent a sustainable concept to maximize a battery's specific energy. However, reactions between polysulfide species and the copper negative electrode can prevent their reversible operation. While this necessitates restricting the mobility of polysulfide species, a less effective migration barrier is herein demonstrated to benefit the overall reversibility.

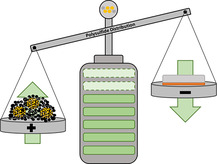

## 1 Introduction

Due to the increasing demands for batteries exceeding energy density and specific energy of the established lithium ion battery technology (LIBs), alternative cell chemistries are required.^[^ ^1^, ^2^, ^3^, ^4^ ^]^ Here, cell concepts referred to as “zero‐excess” or “anode‐free” lithium metal batteries (LMBs), meaning that the positive electrode capacity is deposited on the negative electrode current collector rather than on a pre‐existing reservoir of (excessive) metal upon operation, are considered a promising approach. Compared to conventional LMBs employing lithium metal on a copper current collector as the negative electrode, abandoning metallic lithium and solely using the copper current collector decreases not only costs for raw materials and cell production but also increases gravimetric and especially volumetric energy density.^[^ ^5^, ^6^, ^7^ ^]^ However, without excessive metal replenishing capacity losses upon cell operation, the aforementioned advantages come at the expense of reduced cell longevity.^[^ ^8^ ^]^

While the reversibility of most “anode‐free” LMBs is thought to rely on lithium metal deposition and dissolution at the negative electrode,^[^ ^8^ ^]^ other key performance indicators such as energy density, costs, and sustainability strongly correlate with the choice of positive electrode materials.^[^ ^5^, ^6^, ^7^ ^]^ Lithium–sulfide positive electrodes with specific capacities of up to 1166 mAh g^−1^ and sulfur as an abundant and non‐toxic active material exhibit intriguing properties to construct eco‐friendly batteries with superior energy densities.^[^ ^9^ ^]^ The material utilization and mechanical stability of sulfur‐based positive electrodes however, pose challenges. Furthermore, their intrinsically low electronic conductivity and sluggish kinetics necessitate infiltration into a conductive matrix, limiting the active material content in composite electrodes as well as their rate capability.^[^ ^10^, ^11^, ^12^, ^13^ ^]^ The limited longevity of sulfur‐based positive electrodes is related to the dissolution of polysulfide species, which are intermediate products of the conversion between sulfur and lithium sulfide in ether‐based electrolytes. Their migration to the negative electrode can result in irreversible reactions, consuming active material and thus limiting capacity retention.^[^ ^14^, ^15^, ^16^ ^]^ Though lithium nitrate may diminish the polysulfide shuttle, this electrolyte additive is consumed with consecutive cycling, which renders long‐term retention of polysulfide species at the positive electrode challenging. Furthermore, gaseous products from lithium nitrate decomposition cause cell swelling and may potentially damage the cell housing.^[^ ^17^, ^18^ ^]^ Alternatively, polysulfide shuttling can be addressed via tailored electrolyte formulations with limited polysulfide solubility, which enable quasi solid–solid conversion between sulfur and lithium–sulfide.^[^ ^17^, ^19^, ^20^, ^21^ ^]^ While this has been successfully demonstrated for non‐lithiated positive electrodes comprising elemental sulfur, the larger particle sizes of lithium–sulfide electrodes often result in low accessibility and, in turn, high activation overpotentials.^[^ ^9^, ^22^, ^23^ ^]^ Despite research efforts to decrease the activation overpotential of lithium–sulfide positive electrodes^[^ ^23^ ^]^ via redox mediators^[^ ^24^, ^25^, ^26^ ^]^ or catalysts,^[^ ^27^, ^28^ ^]^ quasi solid–solid conversion for lithium–sulfide positive electrodes remains challenging. With lithiated positive electrodes being required to serve as the capacity reservoir in “anode‐free” cell configurations, dissolution of polysulfide species into the electrolyte is indispensable to activate the positive electrode and enable “anode‐free” lithium–sulfide batteries. Herein, we identify shuttling of polysulfide species to a bare copper negative electrode to be even more detrimental compared to lithium metal negative electrodes, since continuous reactions between polysulfide and the copper current collector can completely prevent the operation of “anode‐free” lithium–sulfide batteries. While utilization of nickel instead of copper foil is a commonly employed^[^ ^26^, ^27^, ^29^ ^]^ yet quite costly^[^ ^30^, ^31^ ^]^ approach to address reactivity between polysulfide species and negative electrodes, we herein utilize electrospinning of a polymer network at the negative electrode^[^ ^32^, ^33^ ^]^ as well as in situ formation of a positive electrode coating^[^ ^34^ ^]^ to limit the migration of polysulfide species toward negative electrodes. While both enable reversible operation of “anode‐free” lithium–sulfide batteries with copper‐based negative electrodes, notable differences in capacity retention can be observed. By extracting and quantifying polysulfide species from different battery components,^[^ ^35^ ^]^ we demonstrate that their distribution throughout “anode‐free” lithium–sulfide batteries is essential to optimize their longevity.

## 2 Results and Discussion

**Figure 1:**
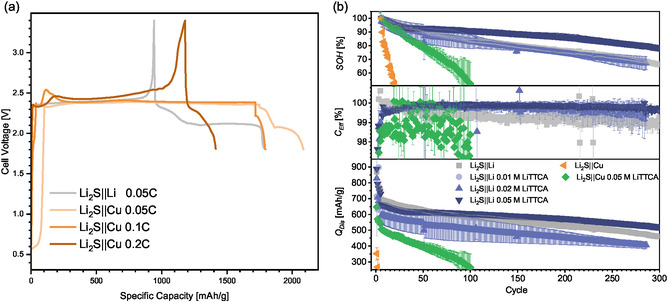
Comparison of coin cells employing Li_2_S positive electrodes and Li and Cu negative electrodes, respectively. a) Initial voltage and b) impact of electrolyte additive LiTTCA on the electrochemical performance (specific discharge capacity *Q*_Dis_, Coulombic efficiency *C*_Eff_, and state of health SOH).

Operating dry‐coated Li_2_S composite positive electrodes with an established liquid electrolyte (1 M lithium bis(trifluoromethanesulfonyl)imide (LiTFSI) + 0.5 M LiNO_3_ in 1,3‐dioxolane (DOL)/1,2–dimethoxyethane (DME) 1:1 Vol:Vol) and 20 μm thin lithium metal on 10 μm copper negative electrodes yields a typical charge–discharge voltage profile. Here, a steady activation plateau indicative of polysulfide formation upon charge and a two‐step discharge process for the reduction of short and long chain polysulfide species can be observed (**Figure** **1**a).^[^ ^21^, ^36^ ^]^ Substitution of lithium metal with a bare copper negative electrode disrupts these processes, displaying a charge plateau with specific capacities well beyond the theoretical capacity of Li_2_S and notably diminished polysulfide reduction capacity upon discharge. While lower specific discharge capacities in the “anode‐free” cell configuration could be reasoned with the absence of an excess lithium metal reservoir, the notably increased charge capacity and missing termination of the process by the upper cut‐off voltage indicate ongoing decomposition reactions. Note that increasing the operating rate and thereby accelerating conversion of polysulfide species into elemental sulfur results in a typical voltage profile upon charge but also yields limited discharge capacities.

Since these phenomena are exclusively observed in “anode‐free” lithium–sulfide batteries, we assume a continuous reaction between polysulfide species and copper, consuming positive electrode active material and thus limiting its relithiation capacity. Immersing pieces of copper foil in polysulfide solutions corroborates this hypothesis, since discoloration of the solution indicates the consumption of polysulfide species. (Figure S1, Supporting Information). Though native passivation layers on copper (Cu^*n+*^) decrease the reaction kinetics relative to acid washed copper solely handled in inert atmosphere (Cu^0^), discoloration of the yellow polysulfide solution and formation of a black solid as the reaction product can be observed over time in both cases. With nickel instead of copper foil, the polysulfide solutions maintain their color independent of passivating surface layers, indicating no reaction and rationalizing nickel‐based “anode‐free” lithium–sulfide batteries in the literature.^[^ ^26^, ^27^, ^29^ ^]^ Since the lower abundance and in turn increased costs of nickel^[^ ^30^, ^31^ ^]^ opposes the concept of affordable and eco‐friendly “anode‐free” lithium–sulfide batteries, approaches to utilize copper‐based negative electrodes, despite their reactivity with polysulfide species, are of high interest.

Employment of localized‐high‐concentrated (LHCE)^[^ ^20^, ^21^ ^]^ or weakly solvating (WSE)^[^ ^17^, ^19^, ^37^ ^]^ electrolytes could enable quasi solid–solid conversion of elemental sulfur to Li_2_S. However, when operated with lithium–sulfide positive electrodes, the obtained specific charge and discharge capacities are well below the capacities with a conventional electrolyte (Figure S2, Supporting Information). Here, limited dissolution of polysulfide species due to either weakly solvating properties in WSE^[^ ^19^, ^21^, ^37^ ^]^ or low availability of uncoordinated solvent molecules in LHCE^[^ ^38^, ^39^ ^]^ only renders fractions of the active material available for electrochemical reactions.^[^ ^22^, ^23^ ^]^ Hence, allowing for polysulfide dissolution but implementing a diffusion barrier is required to operate “anode‐free” lithium–sulfide batteries with copper negative electrodes. A straightforward approach to restrict polysulfide mobility is addition of lithium trithiocyanurate (LiTTCA) as an electrolyte additive, which was previously reported to form a coating at sulfur‐based positive electrodes via in situ polymerization.^[^ ^34^ ^]^ Due to this migration barrier, polysulfide shuttling and active material loss are expected to be diminished. Employing an optimized additive concentration, Li_2_S||Li cells display reduced initial discharge capacities but a superior average Coulombic efficiency (*C*_Eff_) of 99.85% over 300 cycles in (Figure 1b). This enables an almost twofold increase in capacity retention (287 instead of 150 cycles to <80% state of health, (SOH)) compared to reference cells without LiTTCA electrolyte additive. Note that due to improved purity of LiTTCA (Figure S3, Supporting Information) obtained via an alternative synthesis route, the previously optimized additive concentration of 0.1 mol L^−1^^[^ ^34^ ^]^ was not soluble within the electrolyte, yielding 0.05 mol L^−1^ as the optimized additive concentration in our case.

**Figure 2:**
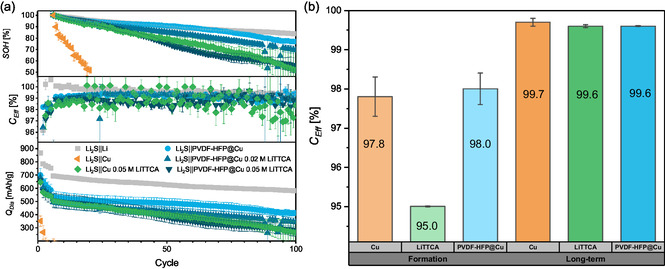
Impact of LiTTCA electrolyte additive and PVDF‐HFP electrospun scaffolds on electrochemical performance in a) “anode‐free” lithium–sulfide batteries and b) Li||Cu cells.

Next to notable improvement of cycle life in conventional lithium–sulfide batteries, retaining polysulfide species at the positive electrode by addition of LiTTCA to the electrolyte also enables the operation of “anode‐free” lithium–sulfide batteries (Figure 1b). Without a lithium metal electrode, losses of active lithium reservoir by interphase or “dead” lithium formation are not replenished in this cell configuration.^[^ ^8^ ^]^ Thus, reduced Coulombic efficiencies (*C*_Eff_) and specific discharge capacities (*Q*_Dis_) are observed for “anode‐free” lithium–sulfide batteries, causing substantially lower capacity retention (48 instead of 150 cycles to <80% SOH). Since previous reports already demonstrated that electrospun polymers retain polysulfide species at sulfur‐based positive electrodes^[^ ^33^, ^40^, ^41^ ^]^ a network of Poly(vinylidenfluorid*‐* *co*‐hexafluorpropylen) (PVDF‐HFP) nanofibers at the negative electrode (Figure S4, Supporting Information) is also applied as a migration barrier for polysulfide species. Compared to the approach of generating an in situ coating at the positive electrode with LiTTCA, "anode‐free" cells with PVDF‐HFP@Cu negative electrodes exhibit an almost twofold increase in capacity retention (88 instead of 48 cycles to <80% SOH, **Figure** **2**a). Previous publications attributed enhanced reversibility with PVDF‐HFP at the negative electrode to its decomposition into lithium fluoride as an solid electrolyte interphase (SEI) constituent with superior passivation,^[^ ^42^, ^43^, ^44^ ^]^ homogenization of electric field gradients due to its high dielectric constant^[^ ^3^, ^45^, ^46^ ^]^ as well as its ability of gelation with the electrolyte,^[^ ^47^ ^]^ which might avoid local electrolyte and lithium nitrate depletion.^[^ ^17^, ^18^ ^]^ While active lithium losses in the initial cycle diminish the achievable discharge capacity compared to Li_2_S||Li cells (700 mAh g^−1^ *Q*_Dis_ instead of 867 mAh g^−1^), the porous polymer scaffold enables similar capacity retention to cells using a lithium metal negative electrode for 70 charge‐discharge cycles (87% SOH). Though comparability to previous publications on Li_2_S||Cu cells^[^ ^48^, ^49^, ^50^, ^51^, ^52^, ^53^, ^54^, ^55^, ^56^ ^]^ is limited due to varying Li_2_S mass loadings, electrolyte to sulfur ratios and operating rates, the herein presented capacity retention of 77% after 100 cycles with PVDF‐HFP@Cu negative electrodes constitutes a notable improvement (Figure S5 and Table S1, Supporting Information). Nevertheless, further enhanced capacity retention has already been demonstrated using gel‐polymer electrolytes in combination with a catalyzing positive electrode architecture.^[^ ^50^ ^]^

**Figure 3:**
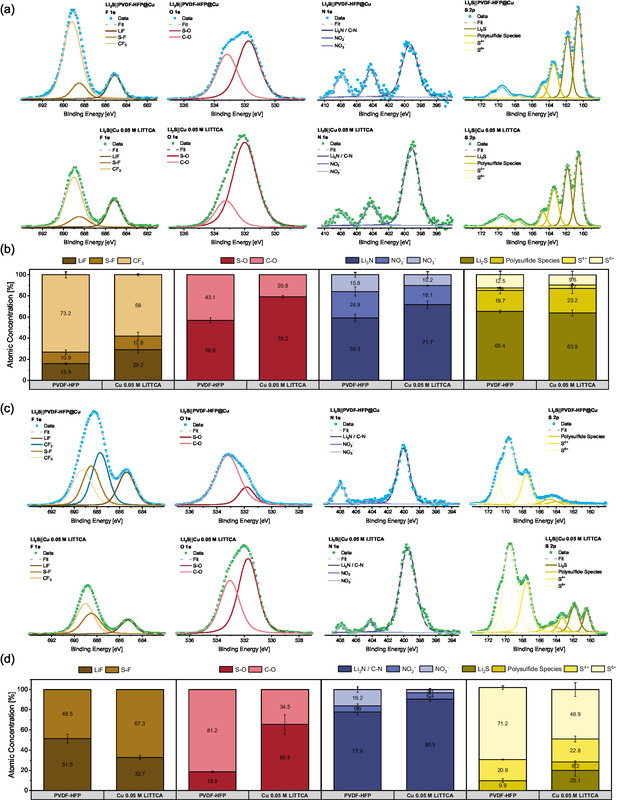
XPS analysis of positive and negative electrodes from Li_2_S||Cu cells with 0.05 M LiTTCA electrolyte additive and Li_2_S||PVDF‐HFP@Cu cells operated with the reference electrolyte after eight charge and discharge cycles. a) Deconvolution of representative F 1s, O 1s, N 1s, and S 2p spectra of positive electrodes. Spectra for the same element but different electrodes are depicted at identical scale to enable a direct comparison of peak intensities. b) Averaged atomic concentrations for the individual species determined in a. Error bars represent the standard deviation between two measurements each on two nominally identical positive electrodes. c) Deconvolution of representative F 1s, O 1s, N 1s, and S 2p spectra of negative electrodes. Spectra for the same element but different electrodes are depicted at identical scale to enable a direct comparison of peak intensities. d) Averaged atomic concentrations for the individual species determined in c. Note that CF_2_ and CF_3_ peaks are neglected in the evaluation due to their presence in PVDF‐HFP. Error bars represent the standard deviation between two measurements each on two nominally identical negative electrodes.

Striving to optimize the achievable capacity retention, both approaches limiting migration of polysulfide species toward copper‐based negative electrodes are combined. However, Li_2_S||PVDF‐HFP@Cu cells with LiTTCA electrolyte additive display diminished capacity retention rather than a symbiotic effect. The reversibility of cell operation is further deteriorated upon increasing LiTTCA concentration in the electrolyte, indicating a negative impact of LiTTCA in “anode‐free” cell configurations. To relate the electrochemical performance of both approaches to chemical compositions of positive and negative electrode–electrolyte interphases, X‐ray photoelectron spectroscopy (XPS) experiments are carried out (**Figure** **3**).

Due to the high abundance of multiple sulfur species at both positive electrodes (Figure 3a), the C—S and S—S bonds previously assigned to polymerized LiTTCA species^[^ ^34^ ^]^ are not identified herein. Nevertheless, F 1s, O 1s, and N 1s spectra all demonstrate a change in positive electrode–electrolyte interphase composition in presence of LiTTCA. Considering that C—N bonds are known to exhibit only slightly higher binding energies than nitrides,^[^ ^34^ ^]^ the increase in Li_3_N/C—N integral, as well as decreased amounts of nitrate‐related species, indicate passivation of Li_2_S electrodes by LiTTCA (Table S2, Supporting Information). This could either originate from polymerization (C—N bonds) or decomposition (potential Li_3_N formation) of the additive. LiTTCA as an additional source of sulfur also increases the amount of S—O species, while notably decreasing the proportion and integral of solvent‐derived C—O species due to its passivating abilities (Table S2, Supporting Information). Similarly, the absolute area (Table S2, Supporting Information) as well as relative fraction (Figure 3b) of LiTFSI‐derived CF_3_ species decrease notably. The integrals for S—F and LiF, however, remain comparable, independent of the electrolyte additive.

For negative electrodes, direct comparison of spectra can be misleading due to differences in macroscopic electrode structure. Empty pores and polymer fibres are expected to dilute the amount of SEI examined during XPS acquisition with PVDF–HFP@Cu negative electrodes, limiting comparability of absolute integrals. Nevertheless, relative concentrations of SEI constituents can be examined when excluding PVDF–HFP‐related signals. Analogous to the positive electrode XPS analysis, incorporation of LiTTCA as an additional source of sulfur and nitrogen also increases the fraction of Li_3_N/C—N species as well as S—O compounds relative to nitrate‐ or solvent‐derived components. Utilizing the porous scaffold results in an increased relative (Figure 3d) as well as absolute (Table S3, Supporting Information) fraction of LiF, which is often detected as a decomposition product of fluoro‐polymers in the vicinity of lithium deposits.^[^ ^42^, ^43^, ^44^ ^]^ This is despite presumably decreased amounts of SEI examined in XPS experiments with scaffold‐decorated negative electrodes. Next to F 1s spectra, the porous scaffold also has a notable impact on chemical nature and amount of sulfur species. While Li_2_S is prominently found within the interphase of bare copper negative electrodes, it is not detected with PVDF‐HFP@Cu negative electrodes. Since Li_2_S originates from the complete reduction of either LiTFSI or polysulfide species from the positive electrode, the electrospun scaffold seems to enhance passivation of lithium metal deposits. Nevertheless, Li_2_S species may still be present within deeper layers of the scaffold and beyond XPS probing depth. To summarize the analysis of positive and negative electrode‐electrolyte interphases, PVDF–HFP scaffolds alter negative electrode passivation, displaying  increased amounts of LiF. Since the electrolyte additive can migrate to positive and negative electrode, LiTTCA‐related species are detected at either electrode. Its passivating ability diminishes decomposition of other electrolyte constituents, especially at the positive electrode. This aligns well with the enhanced capacity retention in the presence of LiTTCA in positive electrode limited Li_2_S||Li cells (Figure 1b). In “anode‐free” cell configurations, however, a detrimental impact on reversibility of lithium inventory can be observed, which may be over‐shadowed by an excessive lithium reservoir in Li_2_S||Li cells.^[^ ^8^ ^]^

To examine whether LiTTCA‐related species detected at negative electrodes impact the reversibility of lithium inventory, electrochemical deposition and dissolution of lithium metal with LiTTCA and PVDF‐HFP@Cu is characterized in Li||Cu and Li||PVDF‐HFP@Cu cells operated with a standardized protocol.^[^ ^57^, ^58^ ^]^ Indeed, additional decomposition reactions of LiTTCA at negative electrodes decrease initial formation cycle reversibility. Nevertheless, all cells exhibit high reversibility of lithium inventory and similar average *C*_Eff_ for repeated deposition and dissolution of lithium metal (Figure 2b). Thus, decomposition of LiTTCA at the negative electrode adds to non‐faradaic reactions within the initial cycle but has no observable negative impact on lithium deposition and dissolution in subsequent charge‐discharge cycles. Since the decreased reversibility of “anode‐free” lithium–sulfide batteries upon addition of LiTTCA to the electrolyte is displayed continuously rather than solely in the initial cycle (Figure 2a), a direct impact of LiTTCA or PVDF‐HFP on lithium metal deposition and dissolution cannot rationalize the observed behavior. Instead, lower reversibility is only observed in presence of a Li_2_S positive electrode, suggesting a correlation to the distribution of polysulfide species. In fact, their presence at negative lithium metal electrodes has been demonstrated to facilitate reactivation of insulated “dead” lithium deposits^[^ ^49^, ^54^ ^]^ and stabilize electrode‐electrolyte interphases.^[^ ^59^, ^60^ ^]^ However, reactions between polysulfide species and active lithium metal or the copper current collector may also cause degradation of active lithium inventory or positive electrode capacity. Thus, PVDF‐HFP and LiTTCA might indeed impact the reversibility of “anode‐free” lithium–sulfide batteries indirectyl by altering the distribution of polysulfide species.

**Figure 4:**
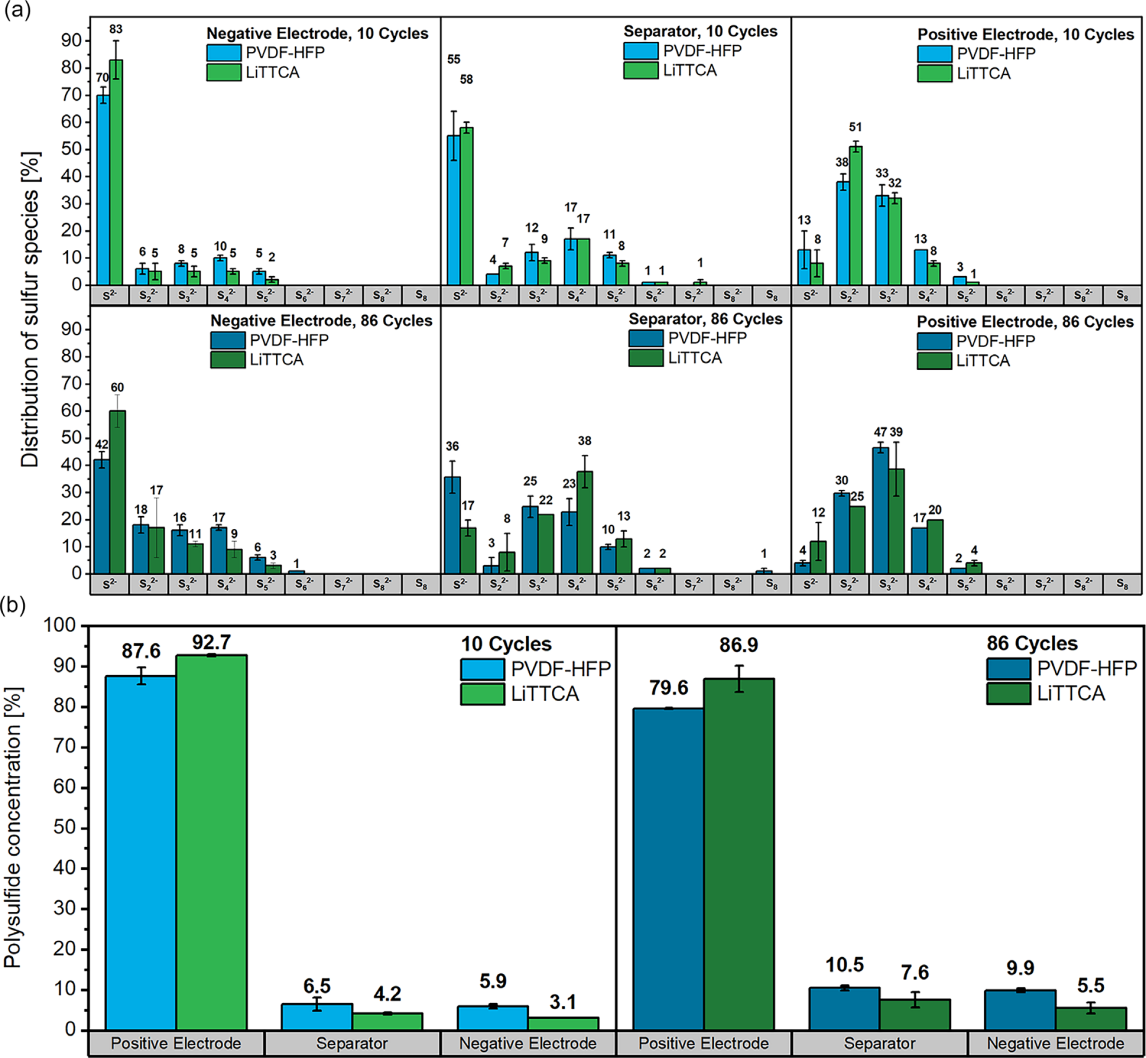
Weight distribution of sulfur species quantified via HPLC‐ICP‐MS technique from extracts of the individual cell components of Li_2_S||PVDF‐HFP@Cu and Li_2_S||Cu cells operated with LiTTCA electrolyte additive. a) Differentiation of chain lengths for the individual cell components. b) Relative polysulfide concentration at the different cell components.

Exploring this hypothesis, cells were disassembled in the discharged state in early stages of cycling (10 cycles) and toward 80% SOH (86 cycles) to extract, separate, and quantify the polysulfide species present in each cell component using a previously published methodology.^[^ ^35^ ^]^ Here, differences in the chain lengths of polysulfide species are observed with both electrospun PVDF‐HFP and LiTTCA electrolyte additive containing cells. In either case, the amount of intermediate polysulfide species (e.g., di‐ and tri‐sulfides) decreases from positive toward negative electrode (**Figure** **4**a). The occurrence of such intermediate species at the positive electrode demonstrates a potential to further enhance its specific capacity by facilitating complete polysulfide reduction to Li_2_S. While neither of the herein applied materials is found to alter the redox processes of polysulfide conversion notably, lower reaction rates (as applied upon cell formation) or catalyzing agents^[^ ^27^, ^28^, ^61^ ^]^ are established approaches to enhance specific discharge capacities. For sulfur species extracted from all negative electrodes, high fractions of monosulfides can be correlated with the reductive properties of lithium metal. Contrary to XPS analysis (Figure 3), Li_2_S are also extracted from PVDF‐HFP@Cu negative electrodes. Thus, they presumably are found in deeper regions of the electrospun scaffold beyond XPS probing depth. Still, bare copper electrodes operated with LiTTCA electrolyte additive display increased fractions of completely reduced sulfide species. In cells disassembled after 86 cycles, an increase in intermediate polysulfide species (chain length 3–5) is observed for every battery component, which is again similar with LiTTCA and PVDF‐HFP@Cu. For positive electrodes, this less complete reduction of polysulfide species after 86 cycles is a potential origin of the displayed decay in specific capacity (Figure 2a). The increase of intermediate polysulfide species at the negative electrode throughout cycling can be rationalized with the accumulation of SEI layers, passivating lithium metal as a reductive agent.

Evaluating the relative sulfur concentration at the individual cell components, notable differences for cells operated with LiTTCA and electrospun PVDF‐HFP@Cu can be observed (Figure 4b, Table S4 and Table S5, Supporting Information). Though the positive electrode contains the highest amount of polysulfide species for either set of cells, fewer (poly)sulfide species are quantified in the separator and at the negative electrode in cells operated with LiTTCA electrolyte additive. Thus,in situ polymerization of LiTTCA is more effective in confining polysulfide species to the positive electrode. This also holds true after 86 cycles, where the largest share of sulfur species is still quantified within the positive electrode with either set of cells, but migration of sulfur species to the negative electrode is notably increased in cells employing electrospun PVDF‐HFP (9.9% versus. 5.5% after 86 cycles). While the limited migration of sulfur species with LiTTCA improves the capacity retention of the positive electrode (Figure 1b), it apparently diminishes the enhanced reversibility at the negative electrode (Figure 2a). Based on previous reports,^[^ ^49^, ^54^, ^59^, ^60^ ^]^ the lower reversibility at the negative electrode can be attributed to less “dead” lithium reactivation from polysulfide species and the lower amounts of sulfur species in the SEI. Thus, LiTTCA as an electrolyte additive boosts the reversibility of conventional lithium–sulfide batteries with an excess lithium reservoir but limits the lifetime of “anode‐free” lithium–sulfide batteries.

Employing PVDF‐HFP@Cu negative electrodes instead of the electrolyte additive, an increased amount of sulfides is quantified at negative electrodes, indicating migration of polysulfide species to be less restricted. In this case, positive electrode stability is sacrificed to achieve higher reversibility of lithium inventory. Adding LiTTCA to the electrolyte, capacity retention of lithium‐sulfide positive electrodes is improved. However, diminished migration of polysulfide species to negative electrodes decreases their reversibility, resulting in a notable deterioration of cycle life (Figure 2a). In other words, a more balanced distribution of polysulfide species between positive and negative electrodes boosts the reversibility of “anode‐free” lithium–sulfide batteries. Still, some restriction of polysulfide migration toward copper negative electrodes is required to reversibly operate “anode‐free’” lithium–sulfide batteries, as demonstrated by ongoing decomposition reactions (Figure S1, Supporting Information).

## 3 Conclusion and Outlook

**Figure 5:**
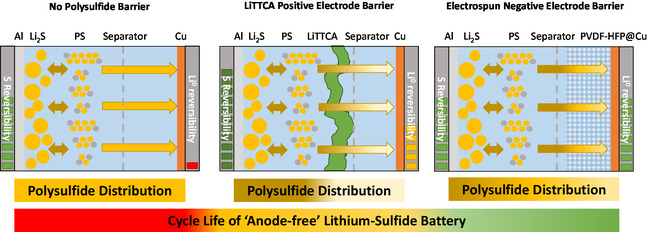
Schematic overview on the proposed correlation of polysulfide distribution and reversibility in “anode‐free” lithium–sulfide batteries.

Within this study, the ambiguous effects of polysulfide migration toward copper‐based negative electrodes in “anode‐free” lithium–sulfide batteries were demonstrated. Due to an ongoing reaction with copper, polysulfide species can be consumed within the initial cycle, completely preventing reversible operation of “anode‐free” lithium–sulfide batteries. Thus, reactions of copper and polysulfide species prior to lithium metal deposition have to be avoided. Constraining migration of polysulfide species toward negative electrodes via an electrolyte additive or electrospun polymeric network enable reversible operation of “anode‐free” lithium–sulfide batteries with copper negative electrodes. However, superior performance of such cells is observed with the latter approach. By individually evaluating electrode reversibilities as well as extracting, separating, and quantifying sulfide species from the different cell components, the importance of tailoring the distribution of polysulfide species and thereby reversibility in “anode‐free” lithium–sulfide batteries is revealed. While the electrolyte additive effectively improves electrochemical stability of the positive electrode due to enhanced passivation and confinement of polysulfide species, reversibility at the negative electrode suffers from their absence, possibly due to less reactivation of “dead” lithium deposits and electrochemically less stable interphase formation. This results in a stabilized positive electrode but too little remaining lithium to completely relithiate it (**Figure** **5**).

With PVDF‐HFP as a more permeable diffusion barrier, a large proportion of polysulfide species still remains at the positive electrode, but their migration towards the negative electrode is less restricted. Increased amounts of polysulfide species at the negative electrode enhance the reversibility of lithium inventory at cost of positive electrode capacity retention, which can be a beneficial compromise for the longevity of cells. This perspective on balancing positive and negative electrode reversibility is crucial to further advance “anode‐free” lithium–sulfide batteries. Boosting the positive electrode stability by limiting migration of polysulfide species only improves overall cell performance of “anode‐free” lithium–sulfide batteries if polysulfide species are not required to enhance negative electrode reversibility. Thus, future efforts should focus on improving reversibility of lithium deposition and dissolution in the absence of polysulfide species first. Here, negative electrode coatings or tailoring of the electrolyte formulation represent promising approaches. Only if the reversibility of lithium inventory outlasts the capacity retention of the positive electrode, strategies that stabilize the positive electrode should subsequently be pursued.

## 4 Experimental Section

4.1

4.1.1

##### Li_2_S Positive Electrodes

Li_2_S positive cathodes were prepared at Fraunhofer IWS using a manual solvent‐free roll process in the glovebox (MBraun, <0.1 O_2_, <0.1 H_2_O) according to carbon‐sulfur composites reported elsewhere.^[^ ^62^ ^]^ The general electrode composition for the electrodes consists of 60 wt% Li_2_S (AMG Lithium), 25 wt% Ketjenblack EC‐600JD (KB), 12 wt% multi‐walled carbon nanotubes (MWCNT, NC7000, Nanocyl), and 3 wt% polytetrafluoroethylene (PTFE) as binder. In a first step, Li_2_S, KB, and MWCNTs were mortared for homogenization. PTFE was added and fibrillated under increased shear forces. To improve formation of PTFE fibrils, the composite was heated up to 100 °C and then formed to a dry‐film between two silicone mats on a hot plate. The lamination of the dry‐film electrode onto the carbon‐coated aluminum (Armor Ensafe91) current collector was carried out at 100 °C. The active mass loading of Li_2_S in these composite electrodes ranged from 1.4–2.9 mg cm^−2^.

##### Copper Electrodes

In a dry room (dew point <−50 °C), a 5 × 10 cm piece of copper foil (Schlenk Metallfolien, 10 μm thickness) was immersed in glacial acetic acid (>99%, Sigma Aldrich). After 10 min, the copper foil was removed from the acetic acid bath. Residual acetic acid was homogeneously evaporated using an argon gas stream. Electrodes punched from copper foil sheets were dried at 100 °C under vacuum overnight.

##### PVDF‐HFP@Cu Electrodes

14 wt% Poly(vinylidenfluorid*‐*co‐hexafluorpropylen) (PVDF‐HFP, Kynar) with an average molecular weight of 675 kg mol^−1^ and a dispersity of 2.0 (determined via gel permeation chromatography with a poly(methylmethylacrylat (PMMA) standard and *N,N*‐dimethylformamid (DMF, VWR Chemicals ≥99.8%) with 0.01 M LiBr as eluent was dissolved in a 3:2 volume ratio mixture of DMF and acetone (Acros Organics, 99+%) by stirring the solution at 70 °C for 2 h. Afterward, this solution was loaded in a 10 mL syringe (Braun inject Luer solo), which was fixed in the syringe pump of the electrospinning device NS 24 (Inovenso). Using a 17 Gauge needle with the prepared syringe, the distance between needle and collector plate, covered with 10 μm thick as‐received copper foil (Schlenk Metallfolien), was set to 17 cm. After establishing a temperature and relative humidity of 25 °C and 25% ± 2%, the electrospinning process was initiated at a flow rate set to 2.3 mL h^−1^ and a voltage of 12 kV. After spinning for 15 min, an electrospun layer with a thickness ranging from 18 μm to 22 μm (Figure S4, Supporting Information), measured using a digital thickness gauge, was obtained. Details on the physiochemical characterization of the electrospun scaffold can be found elsewhere.

##### Electrolytes

The established electrolyte formulation of 1 M LiTFSI and 0.5 M LiNO_3_ in DOL/DME (1:1 Vol:Vol) was obtained by mixing LiTFSI (2.871 g, E‐Lyte Innovations, battery grade) and lithium‐nitrate (LiNO_3_, 0.345 g, E‐Lyte Innovations, battery grade) in 1,3 Dioxolane(DOL, 4.00 g, 3.78 mL, Sigma Aldrich, 99%) and 1,2‐Dimethoxyethane (DME, 3.28 g, 3.78 mL, Sigma Aldrich, 99%, anhydrous). Different amounts of LiTTCA (Synthesis II) were added to this mixture to obtain the respective additive containing electrolyte formulations.

##### Synthesis of LiTTCA: Synthesis I

**Figure 6:**
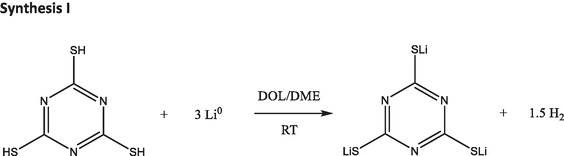
Reaction scheme for the synthesis of LiTTCA according to synthesis route I. Note that the stoichiometric reaction is depicted, but an excess of lithium metal was used.

Following the previously published synthesis route (**Figure** **6**),^[^ ^34^ ^]^ one equivalent of trithiocyanuric acid (TTCA, Sigma Aldrich, 2.5 mmol, 0.443 g) was dissolved in 50 mL DOL (Sigma Aldrich, 99%)/DME (99%, Sigma Aldrich, anhydrous) (7:3 Vol:Vol) in a glovebox (MBraun O_2_ < 0.5 ppm, H_2_O < 0.5 ppm) to obtain a 0.05 M solution. After dissolution of LiTTCA, an excess of metallic lithium (Honjo Lithium, 14.8 mmol, 0.103 g, 6 equivalents) was added to the solution while stirring. Following a color change to yellow and complete consumption of metallic lithium, solvents were removed under reduced pressure (<10^−3^ mbar) at 80 °C. The remaining yellow solid was dried using a turbo pump (Thermo Fischer, <10^−6^ mbar) for 4 days. 20 mg of dried product was dissolved in deuterated dimethyl sulfoxide (DMSO‐d^6^, Sigma Aldrich) to measure ^1^H and ^13^C NMR spectroscopy (Figure S3, Supporting Information).

##### Synthesis II

**Figure 7:**
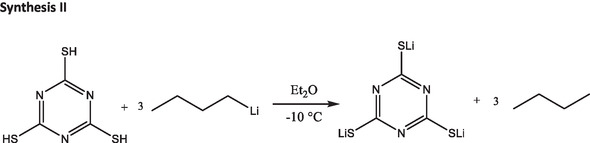
Reaction scheme for the synthesis of LiTTCA according to synthesis route II. Note that the stoichiometric reaction is depicted, but slightly less than 3 equivalents of *n*‐butyl‐lithium were used.

Since NMR spectra of LiTTCA obtained via synthesis route I (Figure S2, Supporting Information) displayed decomposition products of DOL and DME with lithium metal, an alternative synthesis route (**Figure** **7**) was utilized. One equivalent of TTCA (Sigma Aldrich, 2.0 mmol, 0.354 g) was added to a predried Schlenk flask under argon flow and dissolved in distilled diethyl ether (Sigma Aldrich, 99%). After cooling the solution to –10 °C using a sodium chloride ice bath, slightly less than three equivalents of *n*‐butyl‐lithium in hexane (2.5 mol L^−1^, 2.35 mL, 5.8 mmol) was added to the solution, resulting in a color change from transparent to yellow. After stirring at −10 °C for 3 h, a cloudy solution was obtained. Diethyl ether was removed under reduced pressure (<10^−3^ mbar) and the resulting yellow‐white powder was dried using a turbo pump (Thermo Fischer, <10^−6^ mbar) for 4 days. 20 mg of dried product was dissolved in deuterated DMSO‐d^6^ (Sigma Aldrich) to measure ^1^H and ^13^C NMR spectroscopy.

##### Polysulfide Reactivity Test

A 0.04 mol L^−1^ polysulfide solution was synthesized by reacting Li_2_S (0.160 g, 0.6 mmol, 8 equivalents, ABCR, 98%) and elemental sulfur (0.046 g, 1 mmol, 5 equivalents, Sigma Aldrich, 99%) in 25 mL of a 1:1 (Vol:Vol) mixture of DOL (99%, Sigma Aldrich) and DME (99%, anhydrous, Sigma Aldrich) in an argon‐filled glovebox (MBraun, <0.5 O_2_, <0.5 H_2_O). After stirring for 48 h, a brown colored solution was obtained.

To test the reactivity of polysulfide species with copper and nickel foil, 2 mL of the 0.04 mol L^−1^ polysulfide stock solution was diluted by addition of 18 mL of a 1:1 (Vol:Vol) mixture of DOL and DME, yielding 20 mL of a 4 mmol L^−1^ polysulfide solution. As received pieces of copper and nickel foil (Schlenk Metallfolien) containing a native surface passivation layer as well as acid washed copper and nickel foil were immersed in these solutions in an argon‐filled glovebox (MBraun, <0.5 O_2_, <0.5 H_2_O). To produce acid washed copper and nickel foil without a passivation layer, copper and nickel foil were added to a predried and argon‐flushed Schlenk flask. Under an argon atmosphere, glacial acetic acid (Sigma Aldrich, >99%) was added, removing the native surface layer. To avoid reformation of a passivation layer due to exposure to ambient conditions, glacial acetic acid was removed under reduced pressure (<10^−3^ mbar) and the cleaned foils were transferred to the glovebox under argon atmosphere using the Schlenk flask.

##### Cell Assembly

Electrochemical testing of all cell chemistries was carried out in a CR2032 coin cell setup (TOB New Energy). Positive and negative electrodes were separated with a 16 mm Ø separator (Celgard 2500) and sandwiched between a 0.5 mm thick stainless steel spacer (TOB New Energy), a 1.2 mm thick stainless steel wave spring (TOB New Energy), and a 1 mm thick stainless steel spacer (TOB New Energy). Every cell contained 30 μL of electrolyte. For conventional lithium–sulfide batteries, a 14 mm diameter Li_2_S composite electrode was paired with a 15 mm diameter lithium (20 μm thick lithium metal on 10 μm thick copper, Honjo Lithium) electrode. For “anode‐free” lithium–sulfide batteries, a 14 mm diameter Li_2_S composite electrode was paired with a 15 mm diameter bare or PVDF‐HFP decorated copper electrode. In Li||Cu cells, a 12 mm diameter piece of lithium metal (300 μm thickness, Honjo Lithium) served as the positive electrode and was paired with a 15 mm Ø negative electrode. All cells were assembled in an argon‐filled glovebox (MBraun, <0.5 ppm H_2_O, <0.5 ppm O_2_), wetted with 30 μL of the respective electrolyte and sealed using an automated electric crimper (Hohsen Corporation).

##### Cell Testing

Conventional and “anode‐free” lithium–sulfide batteries were operated with an identical testing protocol. In all cycles, 1.8 V was used as the lower cut‐off voltage. To activate the positive electrode, an upper cut‐off voltage of 3.4 V was used in the initial cycle. For further cycling, 2.6 V was set as the upper cut‐off voltage. While the initial five cycles were carried out at 0.05 C (based on 1 C = 1166 mAh/g_Li2S_) charge and discharge current, 0.1 C was used as the charge and discharge current in the following cycles.

For the evaluation of Li||Cu cells, a standardized protocol with a current density of 0.5 mA cm^−2^ was slightly modified.^[^ ^57^, ^58^ ^]^ In the initial formation cycle, lithium was deposited on copper with a capacity of 5 mAh cm^−2^ and dissolved until the cut‐off voltage of −0.1 V was reached. Subsequently, a lithium reservoir of 5 mAh cm^−2^ was deposited on copper. After 10 cycles of repeated 1 mAh cm^−2^ lithium dissolution and deposition, the remaining lithium reservoir was removed until a cut‐off voltage of 0.1 V was reached. The long‐term Coulombic efficiency was calculated by dividing the cumulated lithium dissolution capacity after the formation cycle by the cumulated lithium deposition capacity after the formation cycle.

##### X‐Ray Photoelectron Spectroscopy

XPS experiments were carried out with nanoAnalytics GmbH. The spectrometer (Thermo VG scientific) used an Al‐Kα source (1486.6 eV) and a vacuum sample holder to avoid exposure of electrodes to ambient conditions. Two positive and negative electrodes each were harvested from Li_2_S||PVDF‐HFP@Cu and Li_2_S||Cu coin cells operated with the previously specified protocol for eight charge and discharge cycles, including formation cycles. For each electrode, two different spots were measured, yielding a total of four XPS experiments to be averaged. All spectra were calibrated to a binding energy of 284.8 eV for the C—C bond and fitted using CasaXPS 2.2.36 software.

##### Quantification of Polysulfide Distribution

A previously described technique based on the high‐performance liquid chromatography (HPLC) hyphenated with inductively coupled plasma mass spectrometry (ICP‐MS) was applied to characterize the polysulfide distribution in the electrolyte components.^[^ ^35^ ^]^ After operation, cells were disassembled in an argon‐filled glovebox (MBraun, <0.5 ppm H_2_O, <0.5 ppm O_2_). Separated battery components (negative Cu or PVDF‐HFP@Cu electrodes, separators and positive Li_2_S electrodes) were placed in a 2 mL centrifuge PTFE‐filter (Thermo Scientific), followed by addition of 10.0 μL methyl trifluoromethanesulfonate (>98%, TCI) and 0.50 mL acetonitrile (>99.9%, extra dry, Thermo Scientific) and centrifugation at 14,500 rpm for 4 min (Eppendorf MiniSpin plus).

The resulting extracts were transferred into glass vials and 5.0 μL of each was injected into the HPLC system. For a reversed‐phase chromatographic separation, a Thermo Scientific Dionex UltiMate 3000 series was equipped with a Hypersil GOLD C18 (50 × 2.1 mm, 1.9 μm) column heated at 40 °C. The separation was conducted under isocratic conditions using methanol–water in a 1:1 volume ratio (HPLC‐grade methanol (Merck) and deionized water from a Millipore Milli‐Q water system) with the flow rate of 0.5 mL min^−1^.

Thermo Element R was employed to monitor^[^ ^32^ ^]^ S^+^ signal of the eluate as described in the original method, with the only difference of auxiliary gas flow rate of 1.0 L min^−1^. The weight distributions of sulfur species were calculated based on the peak areas in the resulting chromatograms. For the quantification, a series of aqueous LiTFSI (>99.95%, Sigma Aldrich) solutions with known concentrations was prepared and used as external standards.

## Conflict of Interest

The authors declare no conflict of interest.

## Supporting information

Supplementary Material

## Data Availability

The data that support the findings of this study are available from the corresponding author upon reasonable request.
