# Supplementary material for: Balancing Polysulfide Distribution in “Anode‐Free” Lithium–Sulfide Batteries
Source: ChemSusChem. 2025 Oct 8;18(23):e202501104. doi: 10.1002/cssc.202501104 (PMC12665886; doi:10.1002/cssc.202501104)
Supplement: Supplementary file 1 — Supplementary Material [file CSSC-18-e202501104-s001.pdf]

Supporting Information: Balancing Polysulfide Distribution in 'Anode-Free' Lithium-Sulfide Batteries

Lennart Wichmann[a], Aleksei Sadykov[b,c], Pascal Seete[d,e], Bärbel Tengen[a,c], Peng Yan[a], Tom Boenke[e], Isidora Cekic-Laskovic[a], Sascha Nowak[b], Holger Althues[e], Stefan Kaskel[d,e], Martin Winter[a,b], Gunther Brunklaus[a,*]

a Helmholtz – Institute Münster, IMD-4, Forschungszentrum Jülich GmbH, Corrensstr. 46, 48149 Münster, Germany

b University of Münster, MEET Battery Research Center, Institute of Physical Chemistry, Corrensstr. 46, 48149 Münster, Germany

c University of Münster, International Graduate School for Battery Chemistry, Characterization, Analysis, Recycling and Application (BACCARA), Corrensstr. 40, Münster 48149, Germany

d Technical University Dresden, Chair of Inorganic Chemistry I, Bergstraße 66, 01069 Dresden, Germany

e Fraunhofer IWS, Fraunhofer Institute of Materials and Beam Technology, Winterbergstraße 28, 01277 Dresden, Germany

*Corresponding authors: g.brunklaus@fz-juelich.de (G.B.)

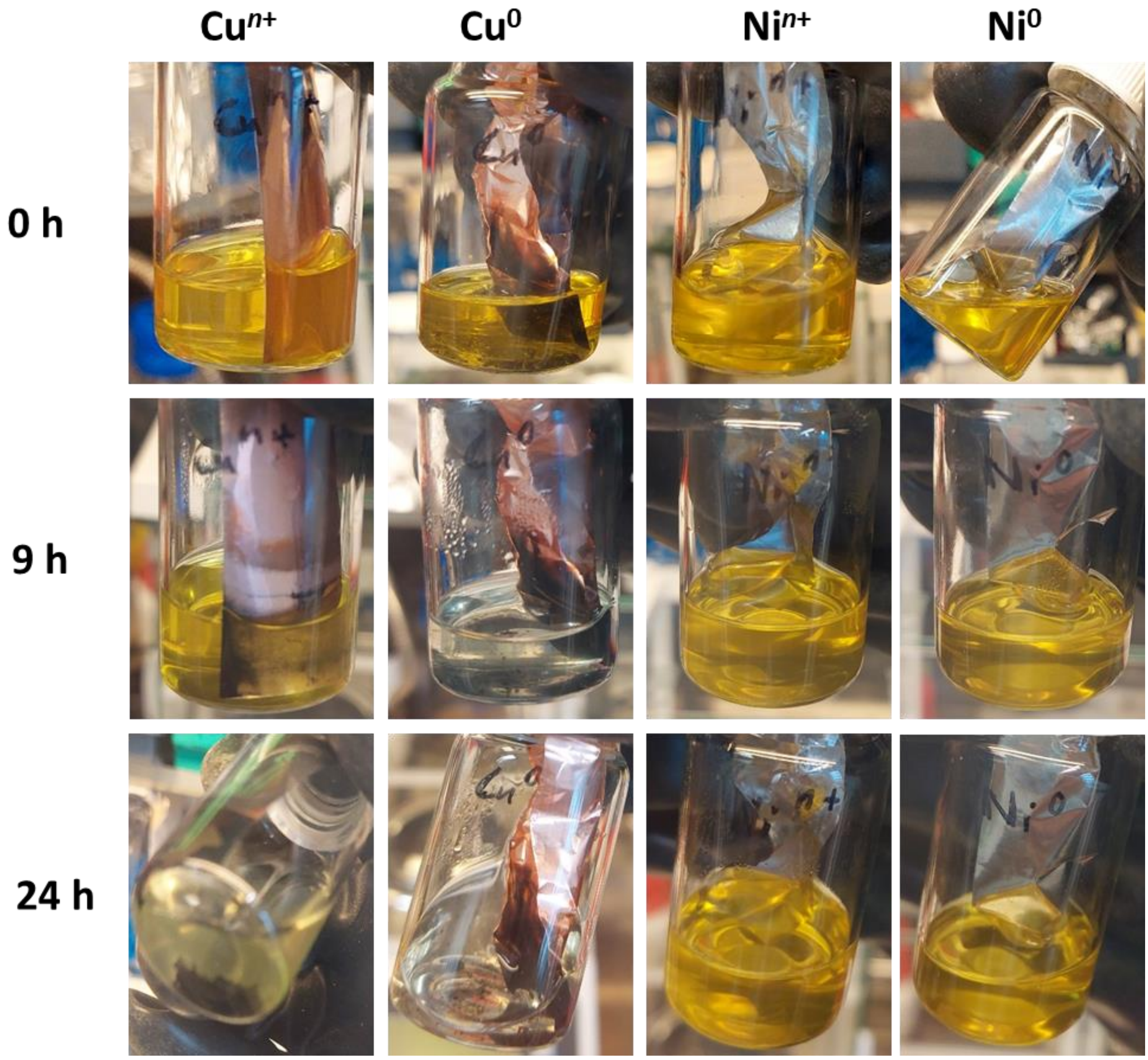

Figure S1: Immersion of copper and nickel foil with a native or acetic acid washed surface layer in polysulfide solution.

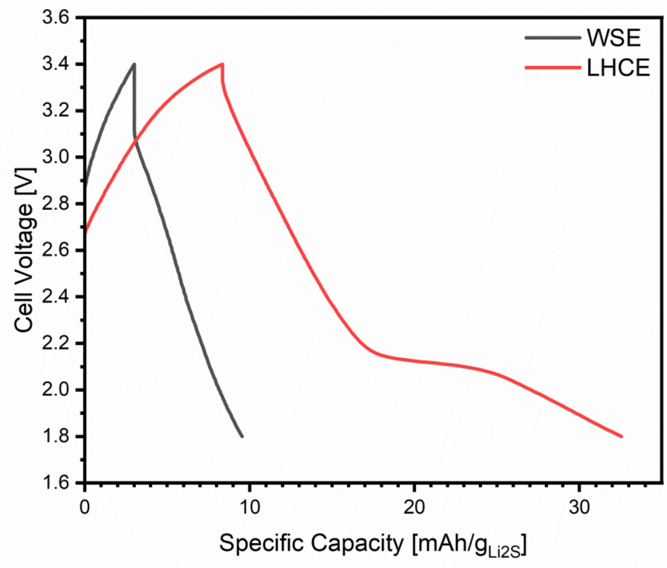

Figure S2: Voltage profiles for the initial cycle of Li_2S||Li cells using a weakly solvating (WSE, 1.4 mol/L LiTFSI in Methoxtrimethylsilane) or localized high concentrated (LHCE, LiFSI:1,2-Dimethoxyethan:1,1,2,2,-Tetrafluoroethyl-2,2,3,3,-tetrafluoropropylether 1 mol : 1.2 mol : 3 mol) electrolyte.

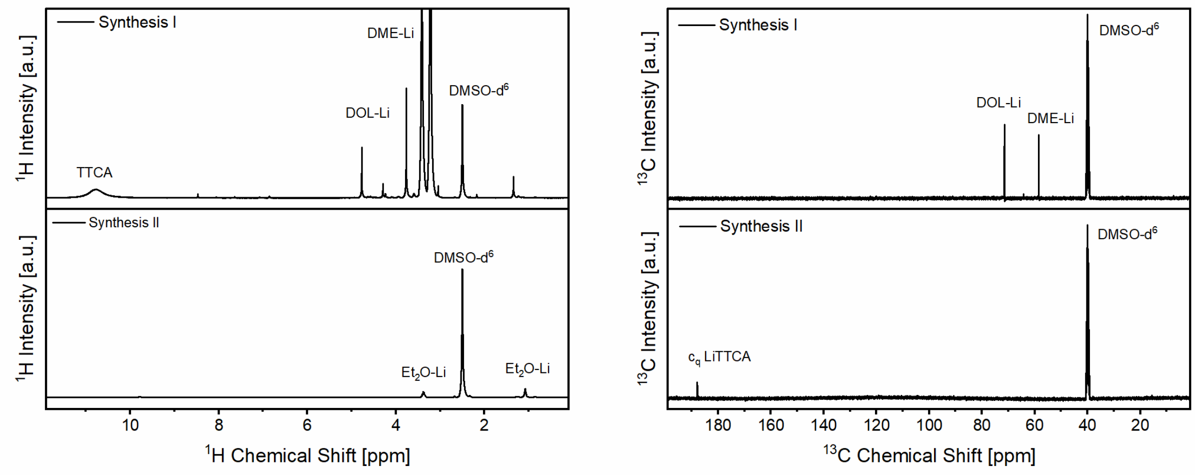

Figure S3: ^{1}H and ^{13}C NMR spectra using different synthesis routes.

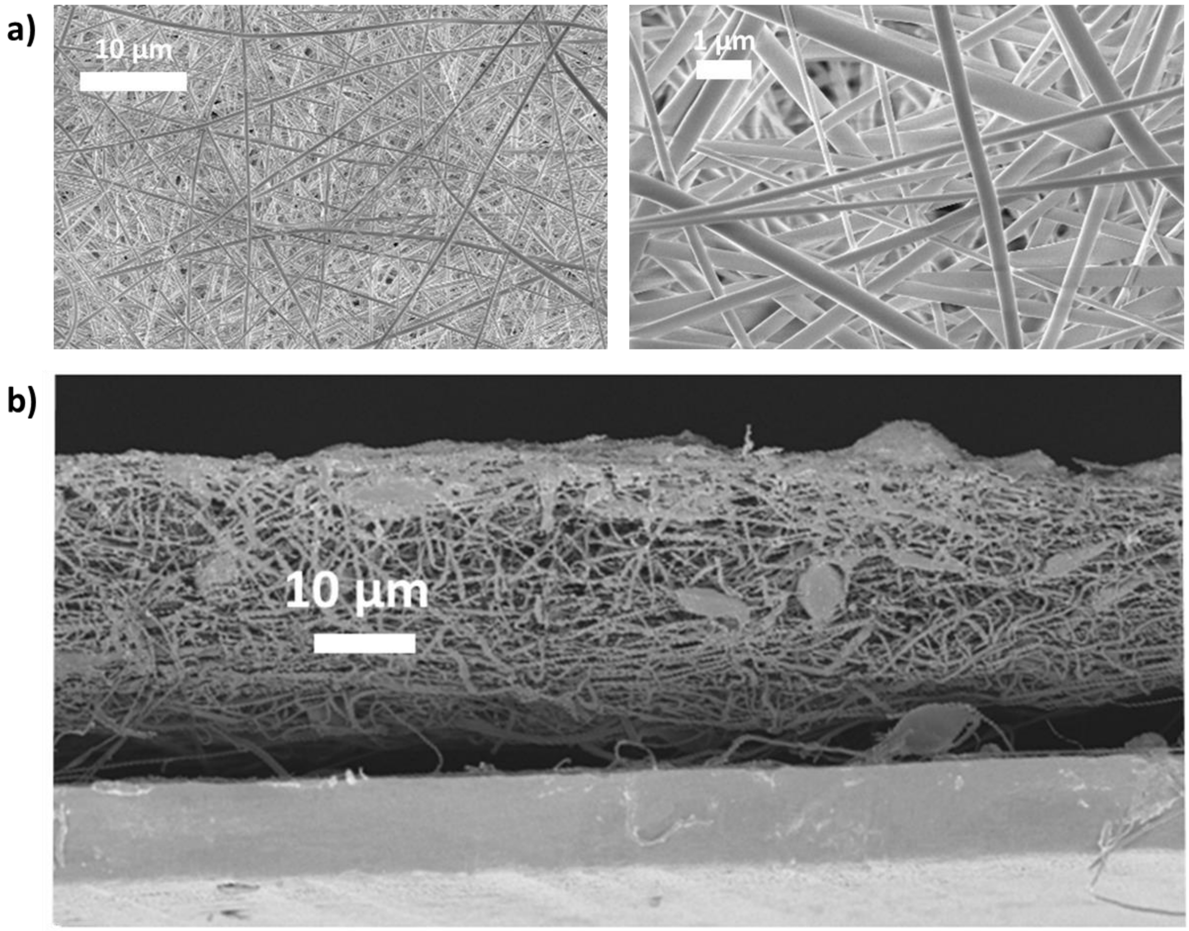

Figure S4: SEM images of electrospun PVDF-HFP@Cu negative electrodes. a) Top view, b) cross-section.

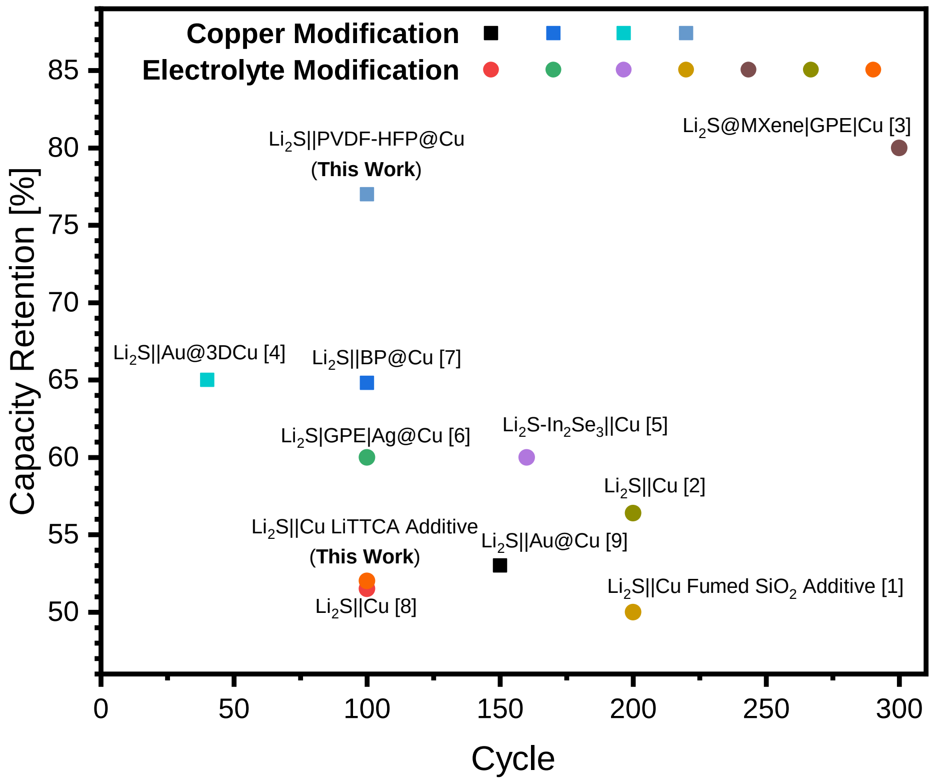

Figure S5: Comparison of capacity retention for Li_2S||Cu cells displayed in this and previous publications [1–9]. In all cases, the best performing cell configuration is depicted.

Table S1: Details for the comparison of Li_2S||Cu cells from this and previous publications [1–9], as depicted in Figure S5.

Ref.	Approach	Li_2S Mass Loading [mg/cm^2]	C-Rate	Cycle	Capacity Retention
1	Fumed SiO_2 Electrolyte Additive	4.0	0.1	200	50%
2	Regular Li_2S||Cu Cell	2.4	0.1	200	56%
3	MXene catalyzed positive electrode + MXene/PVdF-based gel-polymer electrolyte	5.0	0.2	300	80%
4	Ag-coated 3D Cu foam	7.0	0.1	40	65%
5	In_2Se_3 catalyzed positive and negative electrode	4.0	0.2	160	60%
6	Gel-polymer electrolyte + Ag coated Cu	4.2	0.2	100	60%
7	Black phosphorous Cu coating	4.0	0.2	100	65%
8	Regular Li_2S| |Cu Cell	4.0	0.1	100	52%
9	Au-coated Cu	4.0	0.1	150	53%
This work	In situ polymerizing electrolyte additive LiTTCA	2.2	0.1	100	52%
This work	Electrospun PVDF-HFP on copper	2.2	0.1	100	77%
				150	65%
				200	56%

Table S2: Averaged binding energy and raw areas from deconvolution of O 1s, N 1s, F 1s, and S 2p X-ray photoelectron spectra for Li_2S positive electrodes harvested from Li_2S||PVDF-HFP@Cu and Li_2S||Cu cells after 8 charge and discharge cycles. Li_2S||PVDF-HFP@Cu cells were operated with the baseline electrolyte, while Li_2S||Cu cells used the baseline electrolyte with addition of 0.05 M LiTTCA. Errors represent the standard deviation between 4 experiments (2 spots on each of 2 nominally identical electrodes) for both cell chemistries.

	Li_2S Baseline Electrolyte				Li_2S Baseline Electrolyte + 0.05 M LiTTCA			
	Binding Energy [eV]		Area [Arb. Units]		Binding Energy [eV]		Area [Arb. Units]	
Species	Average	Error	Average	Error	Average	Error	Average	Error
CF_3	689.12	0.06	1084	101	689.02	0.01	554	75
S-F	688.50	0.00	166	47	688.45	0.07	127	49
LiF	685.11	0.03	237	38	685.12	0.01	275	12
Li_3N / C–N	399.45	0.09	116	23	399.06	0.07	128	25
NO_2^-	404.25	0.06	51	22	404.16	0.00	33	9
NO_3^-	407.91	0.07	31	7	407.95	0.00	19	10
S-O	531.82	0.09	1100	119	531.95	0.04	1500	198
C-O	533.19	0.08	842	159	533.24	0.07	391	27
Li_2S I	160.54	0.01	512	22	160.57	0.07	466	34
Li_2S II	161.81	0.02	384	17	161.87	0.07	409	30
Polysulfide I	163.47	0.02	206	33	163.44	0.08	246	48
Polysulfide II	164.74	0.02	64	14	164.75	0.09	73	23
S^{4+}	167.51	0.01	32	8	167.55	0.14	20	4
S^{6+} I	169.49	0.00	140	39	169.50	0.09	111	40
S^{6+} II	170.79	0.04	31	9	170.84	0.14	20	4

Table S3: Averaged binding energy and raw areas from deconvolution of O 1s, N 1s, F 1s, and S 2p X-ray photoelectron spectra for negative electrodes harvested from Li_2S||PVDF-HFP@Cu and Li_2S||Cu cells after 8 charge and discharge cycles. Li_2S||PVDF-HFP@Cu cells were operated with the baseline electrolyte, while Li_2S||Cu cells used the baseline electrolyte with addition of 0.05 M LiTTCA. Errors represent the standard deviation between 4 experiments (2 spots on each of 2 nominally identical electrodes) for both cell chemistries.

	PVDF-HFP@Cu				Cu + 0.05 M LiTTCA			
	Binding Energy [eV]		Area [Arb. Units]		Binding Energy [eV]		Area [Arb. Units]	
Species	Average	Error	Average	Error	Average	Error	Average	Error
LiF	685.38	0.00	1586	76	685.24	0.05	524	21
CF_2	687.70	0.05	2129	151	-	-	-	-
S–F	688.48	0.03	1505	218	688.48	0.02	1100	82
CF_3	689.28	0.04	1820	171	689.02	0.02	1227	347
Li_3N / C–N	400.01	0.02	148	7	399.29	0.21	263	38
NO_2^-	404.00	0.00	11	4	404.18	0.03	20	6
NO_3^-	408.00	0.04	31	5	407.89	0.08	10	6
S-O	531.83	0.04	426	18	531.65	0.08	2190	410
C-O	533.20	0.00	1834	33	533.05	0.04	1270	350
Li_2S I	-	-	-	-	160.39	0.04	71	20
Li_2S II	-	-	-	-	161.77	0.09	78	7
Polysulfide I	163.72	0.09	25	7	163.19	0.11	35	6
Polysulfide II	164.69	0.16	28	4	164.55	0.06	21	10
S^{4+}	167.50	0.00	110	2	167.57	0.06	156	22
S^{6+} I	169.57	0.04	286	8	169.46	0.08	297	84
S^{6+} II	170.95	0.05	80	3	170.86	0.04	59	21

Table S4: Sulfur concentrations (per mg of active material used) in the extracts of each cell component of discharged Li_2S||PVDF-HFP@Cu cells disassembled and analyzed in early stages of cycling (10th cycle) and towards reaching 80% state of health (86th cycle).

PVDF-HFP@Cu	10th Cycle				86th Cycle			
	Cell#1	Cell#2	Average	Deviation	Cell#1	Cell#2	Average	Deviation
Positive electrode [ppm/mg$_{Li2S}$]	7353	7445	7399	46	1430	1547	1488	58
Separator [ppm/mg$_{Li2S}$]	404	711	558	154	178	217	197	19
Negative electrode [ppm/mg$_{Li2S}$]	444	557	501	57	186	182	184	2
Total [ppm/mg$_{Li2S}$]	8201	8713	8457	256	1794	1946	1870	76
Positive electrode [%]	89.7	85.4	87.6	2.1	79.7	79.5	79.6	0.1
Separator [%]	4.9	8.2	6.5	1.6	9.9	11.1	10.5	0.6
Negative electrode [%]	5.4	6.4	5.9	0.5	10.4	9.4	9.9	0.5

Table S5: Sulfur concentrations (per mg of active material used) in the extracts of each cell component of discharged Li_2S|| Cu cells operated with 0.05 M LiTTCA as an electrolyte additive. Cells were disassembled and analyzed in early stages of cycling (10th cycle) and towards reaching 80% state of health (86th cycle).

LiTTCA	10th Cycle				86th Cycle			
	Cell#1	Cell#2	Average	Deviation	Cell#1	Cell#2	Average	Deviation
Positive electrode [ppm/mg$_{Li2S}$]	9941	6406	8174	1768	3490	3517	3501	15
Separator [ppm/mg$_{Li2S}$]	483	271	376	105	220	398	309	89
Negative electrode [ppm/mg$_{Li2S}$]	338	216	277	61	159	289	244	65
Total [ppm/mg$_{Li2S}$]	10760	6893	8827	1934	3869	4202	4033	169
Positive electrode [%]	92.4	92.9	92.7	0.3	90.2	83.7	86.9	3.3
Separator [%]	4.5	3.9	4.2	0.3	5.7	9.5	7.6	1.9
Negative electrode [%]	3.1	3.1	3.1	0.0	4.1	6.9	5.5	1.4

Supplementary References

1. Liao, Y. *et al.* Low-cost fumed silicon dioxide uniform Li+ flux for lean-electrolyte and anode-free Li/S battery. *Energy Storage Materials* **48,** 366–374; 10.1016/j.ensm.2022.03.035 (2022).

2. Weret, M. A. *et al.* Reviving Inactive Lithium and Stabilizing Lithium Deposition for Improving the Performance of Anode-Free Lithium–Sulfur Batteries. *ACS Energy Lett.* **8,** 2817–2823; 10.1021/acsenergylett.3c00622 (2023).

3. Liu, Y., Meng, X., Wang, Z. & Qiu, J. Development of quasi-solid-state anode-free high-energy lithium sulfide-based batteries. *Nature communications* **13,** 4415; 10.1038/s41467-022-32031-7 (2022).

4. Cheng, H., Gao, C., Cai, N. & Wang, M. Ag coated 3D-Cu foam as a lithiophilic current collector for enabling Li2S-based anode-free batteries. *Chemical communications (Cambridge, England)* **57,** 3708–3711; 10.1039/D1CC00006C (2021).

5. Zhao, Y., Huang, L., Zhao, D. & Yang Lee, J. Fast Polysulfide Conversion Catalysis and Reversible Anode Operation by A Single Cathode Modifier in Li-Metal Anode-Free Lithium-Sulfur Batteries. *Angewandte Chemie (International ed. in English)* **62,** e202308976; 10.1002/anie.202308976 (2023).

6. Meng, X., Liu, Y., Le Yu, Qiu, J. & Wang, Z. Air-Stable Li 2 S Cathode for Quasi-Solid-State Anode-Free Batteries with High Volumetric Energy. *Adv Funct Materials* **33**; 10.1002/adfm.202211062 (2023).

7. Zhao, Y. *et al.* The beneficial effects of black phosphorous modification of the anode current collector in Li-metal free Li2S-based batteries. *Materials Today Energy* **30,** 101179; 10.1016/j.mtener.2022.101179 (2022).

8. Nanda, S., Gupta, A. & Manthiram, A. A Lithium–Sulfur Cell Based on Reversible Lithium Deposition from a Li 2 S Cathode Host onto a Hostless-Anode Substrate. *Advanced Energy Materials* **8**; 10.1002/aenm.201801556 (2018).

9. Chen, J. *et al.* Li2S-based anode-free full batteries with modified Cu current collector. *Energy Storage Materials* **30,** 179–186; 10.1016/j.ensm.2020.05.009 (2020).
